# Dapsone Resistance in Leprosy Patients Originally from American Samoa, United States, 2010–2012

**DOI:** 10.3201/eid2408.180033

**Published:** 2018-08

**Authors:** Diana L. Williams, Sergio Araujo, Barbara M. Stryjewska, David Scollard

**Affiliations:** Louisiana State University School of Veterinary Medicine, Baton Rouge, Louisiana, USA (D.L. Williams);; Centro de Referência Nacional em Hanseníase/Dermatológica Sanitária, Uberlândia, Brazil (S. Araujo);; National Hansen’s Disease Programs, Baton Rouge, Louisiana, USA (D.L. Williams, S. Araujo, B.M. Stryjewska, D. Scollard)

**Keywords:** leprosy, dapsone resistance, American Samoa, United States, rifampin, National Hansen’s Disease Programs, *folP1*, *rpoB*, tuberculosis and other mycobacteria, *Mycobacterium leprae*, antimicrobial resistance, bacteria

## Abstract

Skin biopsies from US leprosy patients were tested for mutations associated with drug resistance. Dapsone resistance was found in 4 of 6 biopsies from American Samoa patients. No resistance was observed in patients from other origins. The high rate of dapsone resistance in patients from American Samoa warrants further investigation.

Standard treatment for leprosy is multidrug therapy with dapsone, rifampin, and clofazimine (*1*). Resistance to dapsone and rifampin has been observed in many leprosy-endemic regions of the world (*2*–*5*). The Global Sentinel Surveillance for Drug Resistance in Leprosy program was established by the World Health Organization to monitor global leprosy drug resistance among cases of relapse (*6*). With this program, 9 cases of dapsone resistance and 1 case of rifampin resistance were found among 72 leprosy relapse patients from 8 participating countries in 2010 (*7*). However, some leprosy-endemic countries and countries with low incidence, such as the United States (≈200 cases/y), are not involved in this program, even though most US patients with leprosy migrated from endemic areas where drug resistance has been identified (*8*).

Previously, we performed a survey of drug resistance among US leprosy patients referred to the National Hansen’s Disease Programs (Baton Rouge, Louisiana, USA) during 2010–2012 (*8*). Of 39 patients with origins in the Pacific Islands (n = 18), Central or South America (n = 5), Asia (n = 2), and the United States (n = 14), 1 patient had dapsone-resistant *Mycobacterium leprae* and 1 had dapsone- and rifampin-resistant *M. leprae* (*9*). Both of these cases originated in American Samoa.

We expanded this previous survey by evaluating an additional 11 US leprosy patients from American Samoa and US patients from other geographic origins for susceptibility to dapsone and rifampin. All specimens had been referred to the National Hansen’s Disease Programs for histopathologic diagnosis and were tested by using previously published molecular drug susceptibility protocols (*6*). We were able to obtain susceptibility results for only 4 of the 11 US patients from American Samoa. Mutations in *folP*1, a gene associated with dapsone resistance (*4*), were detected in 2 of these patients; no drug resistance was observed in US patients from other origins (Table). Combining these results with our previously published data, 4 of 6 US patients from American Samoa had dapsone-resistant *M. leprae*, and 1 of these was also resistant to rifampin. All 4 dapsone-resistant isolates had distinct mutations (P55L, P55R, T53I, and T53A) in the drug resistance–determining region of the *M. leprae folP*1 gene. The data strongly suggest that these *M. leprae* isolates are not clonal in origin (i.e., did not originate from a single dapsone-resistant clone). In addition, all patients with resistance seemed to have primary resistance to dapsone because biopsies were taken before known treatment with antileprosy drugs.

**Table:** Drug resistance in leprosy patients with and without origins in American Samoa, USA, 2012–2015*

Sample no.	Clinical classification†	Drug susceptibility‡			DRDR sequence		Location§
		Dapsone	Rifampin		*folP*1	*rpoB*	
US patients of American Samoa origin							
NHDP1	BL	No	Yes		T53I	Wild type	Louisiana, USA
NHDP2	LL	Yes	Yes		Wild type	Wild type	Hawaii, USA
NHDP3	LL	No	Yes		P55R	Wild type	Washington, USA
NHDP4	LL	Yes	Yes		Wild type	Wild type	Massachusetts, USA
NHDP5 (*9*)	BL	No	No		T53A	S456L	Hawaii, USA
NHDP6 (*8*)	LL	No	Yes		P55L	Wild type	California, USA
US patients not of American Samoa origin							
NHDP7	BL	Yes	Yes		Wild type	Wild type	Africa
NHDP8	LL	Yes	Yes		Wild type	Wild type	Burma
NHDP9	LL	Yes	Yes		Wild type	Wild type	Micronesia
NHDP10	LL	Yes	Yes		Wild type	Wild type	Micronesia
NHDP11	LL-BL	Yes	Yes		Wild type	Wild type	Micronesia
NHDP12	BL	Yes	Yes		Wild type	Wild type	Micronesia
NHDP13	LL	Yes	Yes		Wild type	Wild type	Pacific Islands
NHDP14	LL	Yes	Yes		Wild type	Wild type	Pacific Islands
NHDP15	LL	Yes	Yes		Wild type	Wild type	Marshall Islands
NHDP16	BL	Yes	Yes		Wild type	Wild type	Marshall Islands
NHDP17	BL	Yes	Yes		Wild type	Wild type	Marshall Islands
NHDP18	BL	Yes	Yes		Wild type	Wild type	The Philippines
NHDP19	LL	Yes	Yes		Wild type	Wild type	The Philippines
NHDP20	BL	Yes	Yes		Wild type	Wild type	Alaska, USA
NHDP21	LL	Yes	Yes		Wild type	Wild type	Alaska, USA
NHDP22	LL	Yes	Yes		Wild type	Wild type	Florida, USA
NHDP23	LL	Yes	Yes		Wild type	Wild type	Florida, USA
NHDP24	LL	Yes	Yes		Wild type	Wild type	Florida, USA
NHDP25	LL-BL	Yes	Yes		Wild type	Wild type	Louisiana, USA
NHDP26	LL	Yes	Yes		Wild type	Wild type	Louisiana, USA

*BL, borderline leprosy; DRDR, drug resistance–determining region; LL, lepromatous leprosy; NHDP, National Hansen’s Disease Programs. †Classification according to the Ridley-Jopling scale. ‡Molecular drug susceptibility testing done according to the World Health Organization guidelines (*6*). A susceptible *Mycobacterium leprae* isolate had no mutations in the DRDR of *folP*1 or *rpoB* (i.e., the isolate was wild type). A drug-resistant *M. leprae* isolate had a mutation in the DRDR of the *folP*1 or *rpoB* gene (*4*,*5*). §Location for patients of American Samoa origin refers to the US state where the leprosy diagnosis was made. Location for patients not of American Samoa origin refers to their location of origin.

The registry of the National Hansen’s Disease Programs indicates that 23 patients from American Samoa were given leprosy diagnoses in the United States during 2002–2014. Because of insufficient DNA or specimen unavailability, drug susceptibility of only 6 patients could be determined. Findings indicate that at least 4 (17%) of these 23 patients were infected with dapsone-resistant *M. leprae*. Biopsy results of 20 US leprosy patients known to be originally from other locations given diagnoses during this time interval did not demonstrate dapsone or rifampin resistance. Eleven (55%) of these patients had Pacific Island origins.

Dapsone resistance in American Samoa could have developed before implementation of multidrug therapy in this population, when dapsone was used as a monotherapy for leprosy. Several patients from American Samoa indicated that frequent visits occur between friends and relatives in American Samoa and Western Samoa; however, no information is currently available regarding drug resistance in Western Samoa.

Dapsone resistance might not necessarily have clinical significance when patients take multidrug therapy as recommended by the World Health Organization. However, in patients with a high bacteria load, resistance to dapsone essentially results in dual therapy with rifampin and clofazimine, placing the burden on just 2 drugs in the multidrug therapy regimen. Moreover, in this regimen, rifampin is taken only once monthly, so the patient is receiving only 1 effective drug (clofazimine) daily. In addition, patient noncompliance might result in the selection of multidrug-resistant *M. leprae*. In another study, resistance to dapsone and rifampin was found in 1 of 4 dapsone-resistant cases, with the 1 case occurring in a relapse patient (*9*). Therefore, *M. leprae* drug resistance (including the identification and evaluation of new markers for dapsone resistance) should be further studied in the American Samoa population (*5*). This research will most likely require correlation of epidemiologic, clinical, and molecular drug susceptibility data from a large number of leprosy patients in this leprosy-endemic region (*10*).
